# Decreased handgrip strength can predict lung function impairment in male workers: a cross sectional study

**DOI:** 10.1186/s12890-020-1135-9

**Published:** 2020-04-20

**Authors:** Makiko Kanai, Osamu Kanai, Kohei Fujita, Tadashi Mio, Masato Ito

**Affiliations:** 1 Panasonic Health Care Center, Panasonic Health Insurance Organization, 5-55 Sotojima-cho, Moriguchi-city, Osaka, 570-0096 Japan; 2 Division of Respiratory Medicine, National Hospital Organization Kyoto Medical Center, 1-1 Fukakusa-Mukaihata-Cho, Fushimi-Ku, Kyoto, 612-8555 Japan

**Keywords:** Spirometry, Hand grip strength, Smoking, Passive smoking

## Abstract

**Background:**

Spirometry is useful for evaluating respiratory health status and predicting health-related outcomes. As spirometry requires skilled technician and takes time, it is useful to find simple way for predicting lung function impairment. The aim of this study was to investigate which tests could predict lung function impairment among workers.

**Methods:**

This prospective study included workers of manufacturing industry who underwent health check-ups in 2017. Subjects underwent the chronic obstructive pulmonary disease (COPD) assessment test (CAT), spirometry, and physical fitness assessments, including handgrip strength (HGS). Lung function impairment was defined as a decline in any of forced expiratory volume in 1 s (FEV1), forced vital capacity (FVC), or a FEV1/FVC ratio less than the lower limit of normal (LLN).

**Results:**

Complete data on 475 workers (366 men, 50.4% ever smokers) were available. Lung function impairment was observed in 99 subjects (64 men). Men with lung function impairment had significantly higher rate of ever-smoking, passive smoking at home in childhood, high CAT scores, and decreased HGS, compared with those without. On multivariate analyses, ever-smoking (odds ratio [OR], 2.50; 95% confidence intervals [CI], 1.25–4.97), passive smoking at home in childhood (OR, 2.71; 95% CI, 1.16–6.32), CAT scores (OR, 1.06; 95% CI, 1.01–1.12), and HGS (OR, 0.73; 95% CI, 0.57–0.92) were independently associated with lung function impairment in men.

**Conclusions:**

Ever-smoking, passive smoking at home in childhood, high CAT scores, and decreased HGS are significantly associated with lung function impairment in men.

**Trial registration:**

Registration number: UMIN000028011. Date of registration: July 1, 2017.

## Background

Lung function impairment, including chronic obstructive pulmonary disease (COPD), is commonly observed in the general population, but it is often undiagnosed [1, 2]. Spirometry is useful not only for evaluating respiratory health status but also for predicting health-related outcomes [3, 4]. Despite this, spirometry is not widely applied in primary care or in health check-ups. This is because the procedure is cumbersome, time-consuming, and tends to be different depending on the ability of the inspector. It would be beneficial to screen subjects who could benefit from spirometry by questionnaire or a simpler examination.

Some simple physical fitness assessments that can be performed in mass screenings have been reported to be associated with a wide range of health-related outcomes. Handgrip strength (HGS) is often used as a low-cost indicator of muscle strength, and it is reported that a lower HGS is associated with a range of health-related outcomes, including all respiratory diseases [5, 6]. The sit-to-stand (STS) test was reported to be strongly associated with mortality in COPD patients [6]. We hypothesized that such simple physical fitness assessments might be predictors of lung function impairment before disease onset in healthy individuals. In the present study, we investigated which factors, including questionnaires, smoking and passive smoking status, and physical fitness assessments, could predict lung function impairment among workers.

## Methods

### Study design

This prospective study included employees of the manufacturing industry in Uji city, Kyoto, Japan, who underwent annual health check-ups, physical fitness assessments and spirometry in July 3–14, 2017. This study was approved by the Ethics Committee of Panasonic Health Care Center (Approval No. 2017–004) and registered in the UMIN Clinical Trials Registry (No. UMIN000028011) on July 1, 2017. All study participants provided their written informed consent and our study adheres to STROBE Statement.

### Measurements

Physical examination included height (m), weight (kg), and blood pressure (mmHg). Hypertension was defined as systolic blood pressure ≥ 140 mmHg or diastolic blood pressure ≥ 90 mmHg or receiving treatment for hypertension. Blood tests were conducted after an overnight fast and included biochemical measurements of triglycerides (TG), low-density lipoprotein cholesterol (LDL-C) and high-density lipoprotein cholesterol (HDL-C), fasting plasma glucose (FPG), glycated hemoglobin (HbA1c), and uric acid (UA). Diabetes mellitus was defined according to American Diabetes Association criteria of FPG ≥ 126 mg/dl, HbA1c ≥ 6.5%, or receiving treatment for diabetes. Dyslipidemia was defined as HDL-C < 40 mg/dl, LDL-C ≥ 140 mg/dl, TG ≥ 150 mg/dl or lipid-specific treatment. Hyperuricemia was defined as UA ≥ 7.0 mg/dl. Information on medical histories, health status, current use of medications, smoking status, and exposure to risk factors (passive smoking and hospitalization due to respiratory diseases in childhood) was obtained using a self-administered questionnaire. All consenting subjects completed a Japanese version of the COPD assessment test (CAT) questionnaire [7]. Smoking status was classified into current, former, and never smokers. For passive smoking, exposure at work (current and previous) and at home (current and in childhood) were asked separately. Ever passive smoking was defined as being exposed currently or previously either at home or at the workplace. Physical fitness assessments included the HGS test and STS test. HGS was measured using a Smedley-type hand dynamometer (TTM Tokyo, Japan). Two attempts were performed with each hand, and the better value of each hand was used for analysis. The STS test was conducted using a standard chair with no arm rests. The subjects were instructed to stand up from and sit down on the chair without using any supports and repeat the procedure as many times as possible in 30 s at a self-selected speed. The number of completed repetitions was recorded [6, 8].

### Spirometry

Forced vital capacity (FVC) and forced expiratory volume in the first second of expiration (FEV1) were measured with a calibrated Chestgraph HI-301 U (CHEST M.I., Inc. Tokyo, Japan) according to the recommended method [9]. The predicted and age-specific lower limit of normal (LLN) FVC, FEV1 and FEV1/FVC were calculated using the equations for the Japanese population [10]. Lung function impairment was defined as decline in FEV1, FVC or FEV1/FVC below the LLN.

### Statistical analyses

Subjects’ characteristics were summarized as number (percentage) for categorical variables and mean (standard deviation) for continuous variables. Comparisons between groups were examined using Fisher’s exact tests for categorical values and Mann-Whitney U tests for continuous values. A *P* value of less than 0.05 was considered statistically significant, and the confidence intervals (CI) were 95%. The STS test and factors with a P value of less than 0.05 in the univariate analyses were included in the multivariate logistic regression analyses. Statistical analyses were performed using R version 3.4.1 (R Foundation for Statistical Computing, Vienna, Austria).

## Results

The study consisted of 475 subjects (366 men and 109 women) who agreed to participate in the research and underwent spirometry and physical fitness assessments. The mean age and standard deviation were 48.9 and 9.2 years old. Current, former and never smokers were 32, 27, and 41% in men and 10, 11, and 79% in women, respectively (Table 1). The prevalence of current exposure to passive smoking at home and in the workplace was 14 and 12% in men and 42 and 2% in women, respectively (*P* < 0.001 and < 0.001). The comorbidity rates of hypertension and hyperuricemia were significantly higher in men than in women (28% versus 17%; *P* = 0.023, and 30% versus 4%; *P* < 0.001, respectively).

**Table 1:** Baseline characteristics

		Overall		Men		Women		*P* value	
n		475		366		109			
Age (years)		48.9	(9.2)	48.4	(9.8)	50.5	(6.4)		0.034
BMI (kg/m^2^)		23.3	(3.8)	23.6	(3.6)	22.09	(4.4)	<	0.001
Smoking status	current	129	(27.2)	118	(32.2)	11	(10.1)	<	0.001
	former	110	(23.2)	98	(26.8)	12	(11.0)		
	never	236	(49.7)	150	(41.0)	86	(78.9)		
Passive smoking	ever passive smoking	404	(85.1)	314	(85.8)	90	(82.6)		0.444
	at home currently	96	(20.2)	50	(13.7)	46	(42.2)	<	0.001
	at home in childhood	351	(73.9)	274	(74.9)	77	(70.6)		0.386
	at workplace currently	47	(9.9)	45	(12.3)	2	(1.8)	<	0.001
	at workplace previously	153	(32.2)	126	(34.4)	27	(24.8)		0.062
Hospitalization due to respiratory diseases in childhood		17	(3.6)	14	(3.8)	3	(2.8)		0.773
Comorbidities									
Hypertension		119	(25.1)	101	(27.6)	18	(16.5)		0.023
Diabetes mellitus		27	(5.7)	21	(5.7)	6	(5.5)	>	0.999
Dyslipidemia		232	(48.8)	187	(51.1)	45	(41.3)		0.081
Hyperuricemia		113	(23.8)	109	(29.8)	4	(3.7)	<	0.001

Data are shown in mean (standard deviation) for continuous values and in number (percentage) for categorical values. *P* values for comparison between sexes are estimated by using Fisher’s exact tests or Mann-Whitney U tests where appropriate

The results of spirometry, CAT scores, and physical fitness assessments are shown in Table 2. Lung function impairment was observed in 18% of men and 32% of women (*P* = 0.002). The mean CAT score was 9.02 in men and 8.29 in women (*P* = 0.223). The HGS of men was significantly higher than that of women (45.2 kg versus 26.1 kg; P < 0.001), while no significant difference was observed in the results of the STS test (29.8 times in men and 28.3 times in women; *P* = 0.036).

**Table 2:** Results of spirometry, COPD assessment test, and physical fitness assessments

n	Overall		Men		Women		*P* value	
	475		366		109			
Spirometry								
FVC (L)	3.73	(0.77)	4.02	(0.59)	2.75	(0.41)	<	0.001
%FVC (%)	92.9	(11.2)	93.6	(11.1)	90.4	(11.3)		0.009
FVC < LLN	70	(14.7)	41	(11.2)	29	(26.6)	<	0.001
FEV1 (L)	3.07	(0.65)	3.31	(0.53)	2.29	(0.35)	<	0.001
%FEV1 (%)	94.9	(12.34)	95.4	(12.4)	93.2	(12.1)		0.094
FEV1 < LLN	68	(14.3)	49	(13.4)	19	(17.4)		0.28
FEV1/FVC (%)	82.6	(5.93)	82.4	(6.02)	83.6	(5.54)		0.052
%FEV1/FVC (%)	102.02	(7.06)	101.73	(7.15)	102.98	(6.69)		0.106
FEV1/FVC < LLN	20	(4.2)	15	(4.1)	5	(4.6)		0.789
Lung function impairment	99	(20.8)	64	(17.5)	35	(32.1)		0.002
Questionnaire								
COPD assessment test	8.85	(5.45)	9.02	(5.31)	8.29	(5.91)		0.223
Physical fitness assessments								
Handgrip strength (kg)	40.78	(10.22)	45.17	(6.60)	26.06	(5.25)	<	0.001
Sit-to-stand test (times)	29.45	(6.80)	29.81	(6.60)	28.25	(7.34)		0.036

Data are shown in mean (standard deviation) for continuous values and in number (percentage) for categorical values. P values for comparisons between sexes are estimated by using Fisher’s exact tests or Mann-Whitney U tests where appropriate

Lung function impairment was defined as a decline in FEV1, FVC or FEV1/FVC less than the LLN. Ever passive smoking was defined as being exposed currently or previously either at home or at the workplace

*FVC* forced vital capacity, *FEV1* forced expiratory volume in 1 s, *LLN* lower limit of normal

**Fig. 1:**
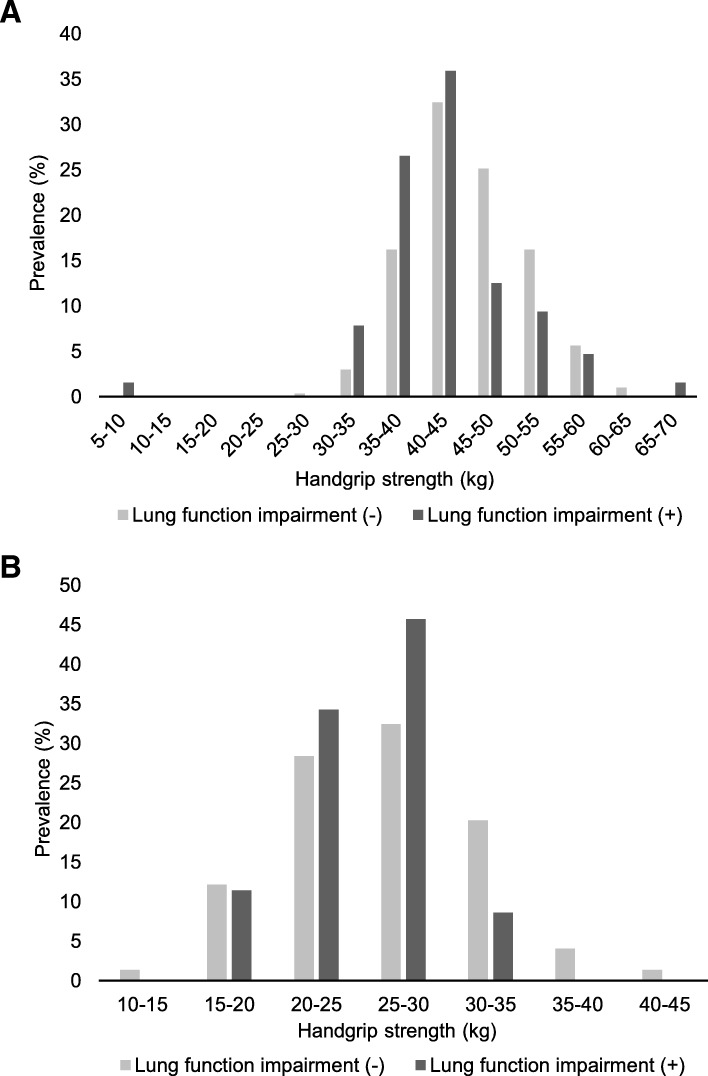
Distribution of handgrip strength classified by the presence of lung function impairment. These histograms show the distribution of handgrip strength in men (**a**) and women (**b**). Bars colored dark gray indicate the prevalence of each levels of handgrip strength in participants with lung function impairment, while bars colored light gray indicate those in participants without lung function impairment

Due to significant differences in baseline characteristics between sexes, we analyzed predictive factors for lung function impairment by sex. Comparisons between men with lung function impairment and those without lung function impairment were significantly different with respect to the following factors (Table 3): ever smoking (73% vs 56%; *P* = 0.011), ever passive smoking (94% vs 84%; *P* = 0.048), passive smoking at home in childhood (89% vs 72%; *P* = 0.004), passive smoking at home currently (22% vs 12%; *P* = 0.045), CAT score (11 vs 8.6; *P* = 0.001), and HGS (43.1 kg vs 45.6 kg; *P* = 0.005). In women, the rate of hospitalization due to respiratory diseases in childhood was higher in those with lung function impairment than in those without (9% vs 0%; *P* = 0.031), while dyslipidemia was more frequent in those without lung function impairment (49% vs 26%; *P* = 0.036). Distribution of HGS by gender are shown in Fig. 1.

**Table 3:** Univariate analyses of predictive factors for any lung function impairment stratified by sex

Sex		Men					Women					
Lung function impairment		No		Yes		*P* value	No		Yes		*P* value	
N		302		64			74		35			
Age (years)		52	[19–62]	53	[19–60]	0.785	52	[21–60]	52	[26–57]		0.896
BMI (kg/m2)		23.1	[17.4–39.0]	23.45	[17.0–40.6]	0.727	22	[16.1–42.3]	20.8	[15.6–30.4]		0.14
Smoking status	ever	169	(56.0)	47	(73.4)	0.011	7	(9.5)	4	(11.4)		0.743
Passive smoking	ever	254	(84.1)	60	(93.8)	0.048	61	(82.4)	29	(82.9)	>	0.999
at home	currently	36	(11.9)	14	(21.9)	0.045	1	(1.4)	1	(2.9)		0.541
	in childhood	217	(71.9)	57	(89.1)	0.004	18	(24.3)	9	(25.7)	>	0.999
while working	currently	34	(11.3)	11	(17.2)	0.209	33	(44.6)	13	(37.1)		0.536
	previously	99	(32.8)	27	(42.2)	0.192	51	(68.9)	26	(74.3)		0.656
Hospitalization due to respiratory diseases in childhood		10	(3.3)	4	(6.2)	0.28	0	(0.0)	3	(8.6)		0.031
Hypertension		78	(25.8)	23	(35.9)	0.123	12	(16.2)	6	(17.1)	>	0.999
Diabetes mellitus		16	(5.3)	5	(7.8)	0.387	5	(6.8)	1	(2.9)		0.662
Dyslipidemia		152	(50.3)	35	(54.7)	0.583	36	(48.6)	9	(25.7)		0.036
Hyperuricemia		88	(29.1)	21	(32.8)	0.551	2	(2.7)	2	(5.7)		0.592
COPD assessment score		8.6	(4.95)	11.0	(6.42)	0.001	7.8	(4.74)	9.4	(7.8)		0.169
Handgrip strength (kg)		45.6	(6.11)	43.1	(8.29)	0.005	26.4	(5.79)	26.37	(3.85)		0.374
Sit to stand test (times)		30.0	(6.58)	28.8	(6.69)	0.2	28.5	(7.31)	27.8	(7.5)		0.644

Data are shown in mean and (standard deviation) for continuous values and in number and (percentage) for categorical values. *P* values for comparisons between respiratory dysfunction or not were estimated by using Fisher’s exact tests or Mann-Whitney U tests where appropriate

We performed initial multivariate analyses adjusted for smoking status, CAT score, HGS, STS test and ever passive smoking (Model 1) and performed secondary analyses adjusted for passive smoking at home currently and in childhood instead of all types of passive smoking (Model 2). Ever smoking, high CAT scores, and decreased HGS were independently associated with lung function impairment in both models (Table 4A). Regarding passive smoking, only passive smoking at home in childhood was significantly associated with lung function impairment (odds ratio, 2.71; 95% confidence intervals, 1.16–6.32; *P* value, 0.021). In women, no significant association was found between predictive factors detected by univariate analyses and lung function impairment (Table 4B).

**Table 4:** Multivariate analyses of predictive factors for lung function impairment

A: Men						
		Model 1			Model 2	
Factors	Odds ratio	95% CI	P value	Odds ratio	95% CI	*P* value
Smoking status (ever versus never)	2.67	(1.35–5.27)	0.0046	2.5	(1.25–4.97)	0.0093
COPD assessment score	1.06	(1.01–1.12)	0.022	1.06	(1.01–1.12)	0.03
Handgrip strength (per 5 kg)	0.74	(0.58–0.94)	0.013	0.73	(0.57–0.92)	0.011
Sit to stand test (per one time)	0.99	(0.94–1.03)	0.51			
Ever passive smoking	2.31	(0.77–6.89)	0.14			
Present passive smoking at home				1.74	(0.83–3.68)	0.15
Passive smoking at home in childhood				2.71	(1.16–6.32)	0.021
B: Women						
Factors	Odds ratio	95% CI	*P* value			
Smoking status (ever versus never)	0.94	(0.31–2.81)	0.91			
Hospitalization due to respiratory diseases in childhood	25,900,000	(0.00 - Inf)	0.99			
Dyslipidemia	0.47	(0.17–1.24)	0.13			
Handgrip strength (per 5 kg)	0.99	(0.91–1.08)	0.78			
Sit to stand test (per one time)	0.97	(0.91–1.04)	0.39			
Odds ratios and *P* values were estimated by using logistic regression analyses adjusted by age, BMI, and smoking history (ever smoking or not). In men, model 1 is adjusted for smoking status, CAT score, hand grip strength, Sit to stand test and all types of passive smoking, while model 2 is adjusted for present and previous passive smoking at home instead of all types of passive smoking. CAT; COPD assessment test, CI; confidence intervals.						

## Discussion

We revealed that decreased HGS was associated with lung function impairment. However, we did not find any significant difference between lung function impairment and the STS test, which is useful for evaluating functional status in patients with COPD [8]. This may result from abundant evidence that low HGS was associated with all-cause mortality and with a wide range of poorer health outcomes, including all respiratory diseases [5, 11]. Regarding COPD, 65.4% of patients with COPD had decreased HGS, and it was associated with respiratory events [12]. Simple functional performance tests, such as the HGS and the STS tests, were reported to be useful for predicting mortality and health-related quality of life in COPD patients [6, 12]. Although there is abundant literature on the association between lung function and HGS, most of them have been studied in the elderly or have investigated the association between disease that has already developed and HGS [13, 14]. In a general population, there are a number individuals with reduced FEV1 and FVC levels, and they have a poorer survival rate than those with normal spirometry results [15]. Therefore, it is important to identify those who can benefit from spirometry before disease onset in general practice. In the present study, odds ratio per 5 kg higher HGS was 0.73 (95% confidence intervals, 0.57–0.92; *P* = 0.0092) for lung function impairment and we showed that HGS was independently associated with lung function impairment among healthy male workers. Our findings suggest that HGS is a useful tool for identifying those with lung function impairment among healthy subjects who will benefit from further respiratory health assessments. Since HGS is an easy, low-cost and reproducible measurement in general practice, it is worthy of further investigation.

In the present study, we demonstrated that both ever smoking and passive smoking at home in childhood could predict lung function impairment in men. Smoking accelerates the decline of FEV1 and is the strongest risk factor for airflow obstruction [16]. Moreover, smoking during adolescence, the late stage of pulmonary growth, impairs both FEV1 and FVC development [17]. Passive smoking is also known to increase the risk of COPD, and subjects reporting previous passive smoking showed significantly declined FEV1 and FVC rates [18]. Longitudinal studies have revealed that the acceleration of the rate of decline in the FEV1 level and early expression of chronic respiratory symptoms are induced by childhood events and exposures in both smokers and nonsmokers [19]. Parental smoking also increases susceptibility to the ill effects for active smokers and affects early lung function deficits in adulthood [20]. In addition to these previous reports, our findings suggest the importance of asking about the history of passive smoking as well as smoking status to identify lung function impairment.

The CAT is a simple questionnaire that was originally developed to measure health-related quality of life and monitor COPD [21, 22]. The total CAT score is reported to have a significant association with a diagnosis of COPD [23, 24]. The CAT is suggested to be useful for screening respiratory health even in an unselected population, and a significant negative correlation between the CAT score and FEV1, FVC, and the FEV/FVC ratio has been reported in the literature [22, 25]. In our study, the CAT score was significantly higher in those with lung function impairment than in those without. The most reliable estimate of the minimum important difference of the CAT is reported as 2 points [26], and the difference observed in our study is considered to have clinical importance. We found that the CAT score was significantly associated with FEV1 < LLN, FVC < LLN, and FEV1/FVC < LLN in men. A higher CAT score may be useful not only for predicting airway obstruction but also for predicting lung function impairment.

There were some limitations. The causal relationship between decreased HGS and lung function impairment is unknown. The present study is a cross-sectional study, and a longitudinal survey is required. Another reason is that there are too many factors contributing to HGS to explain the mechanisms of this relationship. That is, HGS is a marker for nutritional status [27] and is influenced by physical activity [28, 29] and lifestyle factors. In women, we found that HGS was not a significant predictor of lung function impairment. A possible reason for this is that there were not enough subjects to detect a significant difference.

## Conclusions

In the present study, we showed that ever smoking, passive smoking at home in childhood, high CAT scores, and decreased HGS can predict lung function impairment in male workers. Further longitudinal investigation is needed to understand the association of HGS and lung function.

## Data Availability

Because the company in which we conducted the survey has been identified, personal information can be leaked by providing raw data. Therefore, we cannot provide the raw data.

## Abbreviations

BMI: Body mass index
CAT: COPD assessment test
COPD: Chronic obstructive pulmonary disease
FEV1: Forced expiratory volume in 1 s
FPG: Fasting plasma glucose
FVC: Forced vital capacity
HbA1c: Glycated haemoglobin
HGS: Handgrip strength
HDL-C: High-density lipoprotein cholesterol
LDL-C: Low-density lipoprotein cholesterol
LLN: Lower limits of normal
STS: Sit-to-stand
TG: Triglyceride
UA: Uric acid
